# State-Level Lifetime Medical and Work-Loss Costs of Fatal Injuries — United States, 2014

**DOI:** 10.15585/mmwr.mm6601a1

**Published:** 2017-01-13

**Authors:** Feijun Luo, Curtis Florence

**Affiliations:** 1 Division of Analysis, Research and Practice Integration, National Center for Injury Prevention and Control, CDC.

Injury-associated deaths have substantial economic consequences in the United States. The total estimated lifetime medical and work-loss costs associated with fatal injuries in 2013 were $214 billion (*1*). In 2014, unintentional injury, suicide, and homicide (the fourth, tenth, and seventeenth leading causes of death, respectively) accounted for 194,635 deaths in the United States (*2*). In 2014, a total of 199,756 fatal injuries occurred in the United States, and the associated lifetime medical and work-loss costs were $227 billion (*3*). This report examines the state-level economic burdens of fatal injuries by extending a previous national-level study (*1*). Numbers and rates of fatal injuries, lifetime costs, and lifetime costs per capita were calculated for each of the 50 states and the District of Columbia (DC) and for four injury intent categories (all intents, unintentional, suicide, and homicide). During 2014, injury mortality rates and economic burdens varied widely among the states and DC. Among fatal injuries of all intents, the mortality rate and lifetime costs per capita ranged from 101.9 per 100,000 and $1,233, respectively (New Mexico) to 40.2 per 100,000 and $491 (New York). States can engage more effectively and efficiently in injury prevention if they are aware of the economic burden of injuries, identify areas for immediate improvement, and devote necessary resources to those areas.

The numbers of injury-associated deaths in each of the 50 states and DC in 2014 were obtained from the National Vital Statistics System, and state-level lifetime costs were obtained from the Web-based Injury Statistics Query and Reporting System database (*3*). Injury death rates were calculated using the U.S. Census Bureau’s bridged race population estimates for 2014. Lifetime costs, which include lifetime medical and work-loss costs, were computed by multiplying the number of injury deaths by average costs of treating injuries and earnings in 2010, adjusted to 2014 prices. Medical costs were derived from various sources that measure the costs of transport, health care in multiple settings, including emergency departments, hospitals, and nursing homes, and examination by a coroner or medical examiner (*4*). Work-loss costs were developed using earnings data from the U.S. Census Bureau’s Current Population Survey and life expectancy data from CDC’s National Center for Health Statistics. Numbers of deaths, rates, lifetime costs, and lifetime costs per capita (lifetime costs divided by the state population) were examined for each state and DC. Lifetime costs per capita were used for comparisons across states. Four intents of fatal injuries were considered: all intents,* unintentional, suicide, and homicide. For each intent, state-level lifetime costs were estimated for the total population, for males and females, and for all intents. State-level lifetime costs were also estimated for three age groups: young (0–24 years), middle (25–64 years), and older (≥65 years). State-level lifetime costs per capita were provided for the total population for each intent. In some state-intent-population combinations, average medical costs were statistically unstable, but these costs accounted for <1% or <5% of average lifetime costs. When both average medical costs and average work-loss costs were statistically unstable or when the mortality rates were unstable or missing, lifetime costs or lifetime costs per capita were not presented.

## Injuries from All Intents

Injury mortality rates (per 100,000), lifetime costs (in 2014 U.S. dollars), and lifetime costs per capita (in 2014 U.S. dollars) varied widely among the 50 states and DC for each of the four intents. Overall, total injury-related mortality rate and lifetime costs per capita ranged from 101.9 per 100,000 and $1,233, respectively (New Mexico) to 40.2 and $491 (New York) (Table 1). The rates of overall male and female injury mortality were highest in New Mexico (141.1 and 63.7, respectively), and lowest in New York (58.9 and 23.1, respectively). New York also had the lowest injury mortality rate among persons aged ≥65 years (87.1). The states with the highest and lowest lifetime fatal injury costs were California ($20.9 billion) and Vermont ($406 million), respectively. California had the highest number of injury deaths (18,152) and DC the lowest number of injury deaths (385). The lifetime costs per capita for injuries of all intents ranged from $491 to $1,233 (Figure). The five states with the highest lifetime fatal injury costs per capita were New Mexico ($1,233), West Virginia ($1,162), Alaska ($1,091), Louisiana ($1,041), and Oklahoma ($1,040); states with the lowest lifetime costs per capita were New York ($491), New Jersey ($533), California ($538), Massachusetts ($550), and Minnesota ($557).

**TABLE 1:** Deaths from injuries of all intents and unintentional injuries, rates per 100,000 population, lifetime medical and work-loss costs, and lifetime medical and work-loss costs per capita, by state — United States, 2014

State	All intents						Unintentional injuries					
	Total	Sex		Age group (yrs)			Total	Sex		Age group (yrs)		
		Male	Female	0–24	25–64	≥65		Male	Female	0–24	25–64	≥65
**Alabama**												
No. of deaths (rate)	3,625 (73.2)	2,440 (105.3)	1,185 (44.1)	534 (31.6)	2,224 (89.6)	867 (121.9)	2,463 (49.2)	1,525 (65.9)	938 (34.4)	360 (21.5)	1,396 (55.6)	707 (100.5)
Costs, million USD (per capita, USD*)	4,372 (902)	3,317	1,038	983	3,138	163	2,767 (571)	1,967	784	642	1,943	130
**Alaska**												
No. of deaths (rate)	615 (85.8)	441 (119.9)	174 (50.4)	105 (36.3)	427 (106.1)	83 (141.0)	379 (54.9)	260 (74.2)	119 (35.6)	54 (18.5)	259 (64.7)	66 (116.2)
Costs, million USD (per capita, USD*)	804 (1,091)	634	170	193	592	17	448 (608)	341	109	94^a^	349	13
**Arizona**												
No. of deaths (rate)	5,079 (72.6)	3,387 (100.4)	1,692 (45.5)	603 (25.1)	2,900 (85.4)	1,575 (152.7)	3,322 (46.8)	2,077 (61.5)	1,245 (32.5)	357 (14.9)	1,710 (50.0)	1,254 (122.9)
Costs, million USD (per capita, USD*)	5,604 (832)	4,326	1,259	1,129	3,942	260	3,226 (479)	2,425	816	652	2,247	201
**Arkansas**												
No. of deaths (rate)	2,280 (75.2)	1,522 (105.7)	758 (46.4)	316 (30.7)	1,330 (89.6)	634 (140.9)	1,458 (47.2)	907 (62.8)	551 (32.6)	189 (18.4)	757 (50.4)	512 (114.8)
Costs, million USD (per capita, USD*)	2,719 (917)	2,052	642	587	1,904	117	1,623 (547)	1,180	420	347	1,080	91
**California**												
No. of deaths (rate)	18,152 (44.9)	12,820 (66.0)	5,332 (25.0)	2,495 (17.6)	11,109 (52.5)	4,544 (90.2)	11,804 (29.1)	7,847 (40.6)	3,957 (18.3)	1,437 (10.2)	6,832 (32.0)	3,531 (69.8)
Costs, million USD (per capita, USD*)	20,894 (538)	16,746	4,209	4,760	14,766	803	12,171 (314)	9,450	2,808	2,686	8,765	601
**Colorado**												
No. of deaths (rate)	3,883 (72.2)	2,543 (98.1)	1,340 (46.9)	471 (25.3)	2,168 (74.5)	1,243 (193.4)	2,517 (47.1)	1,513 (60.1)	1,004 (34.4)	254 (13.6)	1,221 (41.7)	1,042 (163.2)
Costs, million USD (per capita, USD*)	4,175 (779)	3,202	989	873	2,915	194	2,317 (433)	1,691	640	459	1,602	154
**Connecticut**												
No. of deaths (rate)	2,140 (53.9)	1,373 (76.7)	767 (33.0)	205 (16.6)	1,152 (60.8)	783 (129.5)	1,642 (40.8)	1,005 (56.8)	637 (26.4)	120 (9.7)	824 (44.2)	698 (114.1)
Costs, million USD (per capita, USD*)	2,186 (608)	1,682	464	405	1,584	117	1,446 (402)	1,100	330	234	1,126	100
**Delaware**												
No. of deaths (rate)	629 (65.8)	433 (97.0)	196 (37.4)	79 (25.0)	383 (81.5)	167 (114.8)	425 (43.9)	270 (60.2)	155 (29.1)	50 (15.8)	239 (50.6)	136 (94.8)
Costs, million USD (per capita, USD*)	775 (829)	592	184	146	549	31	472 (505)	334	139	88^†^	338	24
**District of Columbia**												
No. of deaths (rate)	385 (56.2)	264 (81.7)	121 (33.7)	50 (19.8)	250 (67.6)	85 (111.1)	217 (32.7)	130 (42.4)	87 (24.0)	12 (4.8^§^)	133 (36.8)	72 (94.0)
Costs, million USD (per capita, USD*)	479 (726)	370	97	97	357	13	194 (294)	130	61	—^¶^	163	11
**Florida**												
No. of deaths (rate)	13,673 (61.5)	9,216 (88.4)	4,457 (35.8)	1,672 (26.7)	7,363 (71)	4,636 (119.5)	9,433 (41.2)	5,932 (56.2)	3,501 (27)	1,025 (16.5)	4,610 (44.3)	3,796 (97.5)
Costs, million USD (per capita, USD*)	14,763 (742)	11,411	3,326	3,111	9,992	773	9,478 (476)	7,055	2,386	1,859	6,301	608
**Georgia**												
No. of deaths (rate)	6,002 (60.1)	4,061 (85.8)	1,941 (36.5)	946 (25.8)	3,589 (66.8)	1,467 (128.1)	3,964 (40.1)	2,491 (53.8)	1,473 (27.6)	561 (15.4)	2,197 (40.6)	1,206 (106.9)
Costs, million USD (per capita, USD*)	7,055 (699)	5,452	1,582	1,755	4,910	271	4,232 (419)	3,117	1,104	1,009	2,927	214
**Hawaii**												
No. of deaths (rate)	733 (47.3)	527 (70.6)	206 (24.0)	79 (16.1)	428 (57.5)	226 (92.5)	476 (29.9)	327 (43.9)	149 (16.3)	47 (9.6)	246 (32.9)	183 (73.8)
Costs, million USD (per capita, USD*)	825 (581)	684	148	149	588	39	482 (340)	389	86	86	340	30
**Idaho**												
No. of deaths (rate)	1,156 (71.2)	742 (95.2)	414 (48.4)	172 (29.4)	607 (75.0)	377 (172.7)	765 (46.5)	457 (58.8)	308 (35.0)	100 (17.1)	341 (41.6)	324 (149.2)
Costs, million USD (per capita, USD*)	1,274 (780)	916	350	311	814	63	785 (480)	547	238	177	458	52
**Illinois**												
No. of deaths (rate)	6,983 (52.0)	4,808 (75.9)	2,175 (29.6)	1,123 (25.0)	4,006 (58.3)	1,853 (101.7)	4,644 (34.2)	2,918 (46.5)	1,726 (22.9)	557 (12.4)	2,506 (36.3)	1,581 (86.5)
Costs, million USD (per capita, USD*)	8,297 (644)	6,550	1,697	2,104	5,759	311	4,833 (375)	3,579	1,206	1,015	3,502	256
**Indiana**												
No. of deaths (rate)	4,462 (66.5)	3,007 (94.1)	1,455 (40.1)	687 (29.0)	2,685 (79.9)	1,088 (115.6)	2,974 (43.8)	1,853 (58.3)	1,121 (30.0)	390 (16.6)	1,665 (49.2)	919 (97.3)
Costs, million USD (per capita, USD*)	5,240 (794)	4,059	1,190	1,288	3,718	196	3,166 (480)	2,339	823	716	2,279	160
**Iowa**												
No. of deaths (rate)	2,045 (58.4)	1,300 (81.2)	745 (37.4)	237 (21.5)	936 (58.4)	872 (161.7)	1,517 (41.9)	898 (55.8)	619 (29.3)	135 (12.4)	586 (36.3)	796 (146.9)
Costs, million USD (per capita, USD*)	1,987 (639)	1,493	479	435	1,242	140	1,292 (416)	937	343	243	756	123
**Kansas**												
No. of deaths (rate)	1,987 (65.2)	1,292 (89.8)	695 (41.7)	266 (24.7)	1,046 (71.2)	675 (154)	1,377 (44.1)	829 (57.4)	548 (31.5)	157 (14.6)	634 (42.7)	586 (132.3)
Costs, million USD (per capita, USD*)	2,223 (765)	1,697	505	491	1,438	115	1,367 (471)	1,004	339	284	855	97
**Kentucky**												
No. of deaths (rate)	3,634 (80.7)	2,466 (114.5)	1,168 (48.8)	427 (27.8)	2,343 (102.7)	864 (138.4)	2,622 (58.3)	1,677 (78.8)	945 (39.1)	296 (19.3)	1,616 (71)	710 (114.9)
Costs, million USD (per capita, USD*)	4,296 (973)	3,300	1,010	767	3,314	164	2,966 (672)	2,196	775	523	2,293	131
**Louisiana**												
No. of deaths (rate)	3,654 (77.5)	2,576 (113.8)	1,078 (43.7)	659 (39.6)	2,334 (95.9)	659 (107.8)	2,344 (49.6)	1,584 (70.5)	760 (30.4)	381 (22.9)	1,440 (58.9)	522 (86.1)
Costs, million USD (per capita, USD*)	4,839 (1,041)	3,805	1,008	1,233	3,430	127	2,855 (614)	2,203	666	685	2,045	97
**Maine**												
No. of deaths (rate)	952 (65.0)	633 (93.4)	319 (38.5)	105 (26.5)	492 (71.3)	354 (146.8)	690 (45.9)	429 (63.1)	261 (30.0)	63 (15.9)	320 (46.7)	306 (126.7)
Costs, million USD (per capita, USD*)	960 (722)	736	215	200^a^	649	60	626 (470)	467	152	117^†^	423	50
**Maryland**												
No. of deaths (rate)	3,482 (56.1)	2,426 (83.7)	1,056 (31.0)	462 (22.6)	2,129 (65.9)	891 (109.3)	1,674 (26.4)	1,046 (36.9)	628 (17.3)	183 (9)	772 (23.3)	719 (88.3)
Costs, million USD (per capita, USD*)	4,233 (708)	3,376	838	888	3,049	149	1,560 (261)	1,183	363	340	1,039	114
**Massachusetts**												
No. of deaths (rate)	3,452 (47.4)	2,361 (70.4)	1,091 (26.4)	335 (13.8)	2,132 (59.4)	984 (92.1)	2,692 (36.8)	1,767 (53.2)	925 (21.9)	230 (9.4)	1,577 (44.3)	884 (82.4)
Costs, million USD (per capita, USD*)	3,707 (550)	3,032	711	648	2,936	158	2,508 (372)	2,059	503	444	2,143	138
**Michigan**												
No. of deaths (rate)	6,652 (63.8)	4,392 (89.2)	2,260 (39.9)	967 (27.6)	3,807 (74.2)	1,878 (122.5)	4,422 (41.5)	2,714 (55.0)	1,708 (28.9)	532 (15.4)	2,283 (43.9)	1,607 (104.6)
Costs, million USD (per capita, USD*)	7,539 (761)	5,766	1,749	1,780	5,194	322	4,338 (438)	3,168	1,172	943	3,014	264
**Minnesota**												
No. of deaths (rate)	3,226 (54.3)	1,956 (71.6)	1,270 (37.4)	361 (19.6)	1,465 (50.0)	1,400 (168.8)	2,385 (39.2)	1,327 (49.0)	1,058 (29.8)	197 (10.7)	888 (30.3)	1,300 (155.9)
Costs, million USD (per capita, USD*)	3,041 (557)	2,227	771	670	1,953	210	1,855 (340)	1,296	526	358	1,137	190
**Mississippi**												
No. of deaths (rate)	2,477 (81.8)	1,702 (120.0)	775 (47.4)	443 (40.7)	1,421 (93.4)	612 (149)	1,712 (56.2)	1,085 (77.0)	627 (37.8)	288 (26.9)	903 (58.7)	520 (127.4)
Costs, million USD (per capita, USD*)	2,872 (959)	2,306	601	807	1,963	110	1,816 (607)	1,379	447	512	1,215	92
**Missouri**												
No. of deaths (rate)	4,672 (74.1)	3,142 (105.9)	1,530 (43.9)	675 (32.2)	2,658 (85.7)	1,339 (143.6)	3,110 (48.5)	1,911 (64.3)	1,199 (33.4)	407 (19.6)	1,585 (50.9)	1,118 (119.7)
Costs, million USD (per capita, USD*)	5,371 (886)	4,213	1,159	1,249	3,767	230	3,203 (528)	2,379	830	731	2,185	186
**Montana**												
No. of deaths (rate)	902 (83.1)	586 (110.5)	316 (56.2)	121 (34.9)	475 (91.4)	306 (183.8)	581 (52.6)	343 (64.8)	238 (40.5)	75 (21.8)	253 (49.1)	253 (153.3)
Costs, million USD (per capita, USD*)	973 (950)	729	239	219	653	55	579 (566)	411	160	133	359	44
**Nebraska**												
No. of deaths (rate)	1,116 (56.0)	752 (80.6)	364 (32.9)	161 (23.7)	563 (58.9)	392 (134.7)	781 (38.2)	492 (52.8)	289 (24.7)	95 (13.9)	338 (35.1)	348 (118.5)
Costs, million USD (per capita, USD*)	1,139 (605)	913	245	296	754	61	697 (370)	543	169	172	446	52
**Nevada**												
No. of deaths (rate)	1,948 (67.0)	1,359 (94.6)	589 (39.7)	251 (26.6)	1251 (81.2)	446 (121.7)	1,166 (40.1)	750 (52.1)	416 (28.1)	144 (15.3)	722 (46.4)	300 (83.6)
Costs, million USD (per capita, USD*)	2,294 (808)	1,781	534	464	1,665	85	1,319 (465)	975	359	265	949	54
**New Hampshire**												
No. of deaths (rate)	1,001 (70.8)	645 (97.9)	356 (44.6)	92 (20.1)	584 (84.9)	325 (154.8)	716 (50.4)	435 (67.2)	281 (34.2)	56 (12.1)	376 (55.7)	284 (135.6)
Costs, million USD (per capita, USD*)	1,022 (771)	800	226	174	798	55	664 (500)	515	156	104	522	46
**New Jersey**												
No. of deaths (rate)	4,210 (44.4)	2,881 (65.2)	1,329 (25.4)	555 (18.8)	2,454 (51.1)	1,200 (88.4)	2,970 (30.8)	1,935 (43.9)	1,035 (19.1)	319 (10.8)	1,597 (33.1)	1,053 (77.2)
Costs, million USD (per capita, USD*)	4,765 (533)	3,806	961	1,074	3,465	201	2,991 (335)	2,368	657	607	2,238	171
**New Mexico**												
No. of deaths (rate)	2,163 (101.9)	1,443 (141.1)	720 (63.7)	291 (38.5)	1,303 (124.7)	569 (185.9)	1,534 (71.9)	958 (94.3)	576 (49.9)	173 (22.8)	899 (85.7)	462 (152.4)
Costs, million USD (per capita, USD*)	2,573 (1,233)	1,965	603	542	1,844	101	1,659 (796)	1,214	445	315	1,250	79
**New York**												
No. of deaths (rate)	8,585 (40.2)	5,801 (58.9)	2,784 (23.1)	1,046 (15.1)	4,934 (45.9)	2,600 (87.1)	5,945 (27.5)	3,799 (38.8)	2,146 (17.2)	587 (8.5)	3,095 (28.7)	2,259 (75.5)
Costs, million USD (per capita, USD*)	9,689 (491)	7,594	1,987	1,987	6,858	436	5,772 (292)	4,443	1,302	1,095	4,158	363
**North Carolina**												
No. of deaths (rate)	6,541 (63.7)	4,358 (90.8)	2,183 (39.2)	890 (25.6)	3,709 (71.0)	1,940 (140.2)	4,558 (44.3)	2,881 (60.9)	1,677 (29.5)	552 (16.0)	2,378 (45.4)	1,626 (118.7)
Costs, million USD (per capita, USD*)	7,310 (735)	5,674	1,607	1,681	5,148	334	4,620 (465)	3,517	1,093	1,021	3,255	270
**North Dakota**												
No. of deaths (rate)	514 (64.1)	353 (89.5)	161 (38.6)	82 (27.0)	258 (68.6)	174 (149.1)	349 (42.8)	219 (56.8)	130 (29.4)	44 (14.6)	146 (39.3)	159 (135.8)
Costs, million USD (per capita, USD*)	545 (737)	447	100	158^†^	367	30	312 (422)	245	69	82^†^	205	26
**Ohio**												
No. of deaths (rate)	8,366 (69.4)	5,541 (97.9)	2,825 (42.9)	984 (24.8)	5,062 (85.5)	2,320 (128.0)	6,178 (50.6)	3,828 (68.0)	2,350 (34.6)	576 (14.5)	3,595 (60.6)	2,007 (110.6)
Costs, million USD (per capita, USD*)	9,370 (808)	7,217	2,143	1,820	7,038	403	6,200 (535)	4,607	1,609	1,041	4,874	338
**Oklahoma**												
No. of deaths (rate)	3,522 (88.8)	2,277 (119.9)	1,245 (59.6)	485 (34.6)	2,069 (104.3)	968 (176.8)	2,421 (60.3)	1,465 (77.3)	956 (44.5)	283 (20.3)	1,308 (65.0)	830 (152.5)
Costs, million USD (per capita, USD*)	4,035 (1,040)	3,024	981	893	2,841	171	2,508 (647)	1,812	686	511	1,747	141
**Oregon**												
No. of deaths (rate)	2,773 (64.1)	1,805 (88.6)	968 (40.8)	286 (22.1)	1,477 (69.0)	1,010 (161.8)	1,803 (40.8)	1,072 (52.7)	731 (29.5)	156 (12.1)	826 (38.3)	821 (131.8)
Costs, million USD (per capita, USD*)	2,704 (681)	2,075	624	530	1,932	159	1,504 (379)	1,111	383	285	1,068	122
**Pennsylvania**												
No. of deaths (rate)	9,224 (66.1)	6,111 (94.1)	3,113 (40.0)	1,102 (25.4)	5,245 (78.8)	2,875 (127.4)	6,640 (46.6)	4,091 (63.0)	2,549 (31.5)	683 (15.8)	3,454 (52.1)	2,503 (109.7)
Costs, million USD (per capita, USD*)	10,089 (789)	7,874	2,229	2,085	7,225	477	6,420 (502)	4,820	1,633	1,256	4,687	404
**Rhode Island**												
No. of deaths (rate)	748 (62.8)	475 (88.8)	273 (40.0)	59 (15.2)	422 (75.6)	267 (143.4)	592 (49.0)	360 (67.9)	232 (32.7)	33 (8.6)	316 (57.0)	243 (129.3)
Costs, million USD (per capita, USD*)	771 (731)	576	179	113	578	41	526 (498)	387	134	62^†^	420	36
**South Carolina**												
No. of deaths (rate)	3,608 (72.0)	2,422 (103.1)	1,186 (44.0)	564 (33.8)	2,111 (83.4)	933 (132.2)	2,436 (48.2)	1,519 (65.0)	917 (33.4)	334 (20.3)	1,333 (52.1)	769 (110.0)
Costs, million USD (per capita, USD*)	4,279 (885)	3,309	962	1,054	2,925	169	2,693 (557)	1,984	695	615	1,821	136
**South Dakota**												
No. of deaths (rate)	642 (71.1)	415 (97.4)	227 (45.9)	110 (35.9)	320 (75.8)	212 (149.9)	462 (49.2)	282 (65.6)	180 (34.3)	67 (22.1)	195 (45.6)	200 (139.9)
Costs, million USD (per capita, USD*)	687 (805)	505	172	197	448	35	422 (495)	302	111	119^†^	270	31
**Tennessee**												
No. of deaths (rate)	5,237 (77.4)	3,489 (110.5)	1,748 (47.2)	631 (27.9)	3,093 (90.2)	1,512 (163.1)	3,781 (55.5)	2,361 (75.3)	1,420 (37.6)	361 (16.0)	2,116 (61.4)	1,304 (141.7)
Costs, million USD (per capita, USD*)	5,947 (908)	4,556	1,396	1,162	4,262	273	3,900 (595)	2,871	1,030	650	2,843	228
**Texas**												
No. of deaths (rate)	14,652 (55.6)	10,164 (79.8)	4,488 (32.8)	2,454 (24.4)	8,777 (62.2)	3,419 (115.9)	9,723 (37.2)	6,398 (51.2)	3,325 (24.2)	1,498 (14.9)	5,434 (38.3)	2,789 (95.4)
Costs, million USD (per capita, USD*)	17,522 (650)	13,869	3,740	4,549	12,340	615	10,648 (395)	8,237	2,512	2,720	7,485	486
**Utah**												
No. of deaths (rate)	1,924 (73.0)	1,265 (97.1)	659 (49.7)	286 (23.5)	1,190 (85.7)	446 (158.9)	1,167 (45.3)	726 (57.5)	441 (33.5)	141 (11.5)	662 (47.6)	364 (130.0)
Costs, million USD (per capita, USD*)	2,362 (803)	1,794	564	525	1,726	78	1,251 (425)	937	315	250	942	61
**Vermont**												
No. of deaths (rate)	478 (68.2)	291 (91.0)	187 (45.4)	54 (24.6)	208 (64.2)	216 (207.0)	322 (44.4)	168 (53.3)	154 (34.9)	25 (10.9)	112 (34.5)	185 (179.2)
Costs, million USD (per capita, USD*)	406 (648)	314	88	102^†^	265	32	228 (365)	161	62	46^†^	140	27
**Virginia**												
No. of deaths (rate)	4,701 (54.7)	3,141 (77.2)	1,560 (33.7)	634 (21.9)	2,618 (57.9)	1,449 (132.9)	3,147 (36.7)	1,962 (49.2)	1,185 (25.2)	362 (12.5)	1,577 (34.9)	1,208 (111.6)
Costs, million USD (per capita, USD*)	5,166 (620)	3,996	1,128	1,196	3,655	244	3,004 (361)	2,265	720	671	2,163	194
**Washington**												
No. of deaths (rate)	4,428 (59.6)	2,909 (81.9)	1,519 (38.2)	530 (22.0)	2,446 (63.3)	1,451 (149.5)	2,997 (39.9)	1,821 (51.8)	1,176 (28.8)	304 (12.6)	1,451 (37.0)	1,242 (128.6)
Costs, million USD (per capita, USD*)	4,600 (651)	3,550	1,052	1,004	3,262	240	2,727 (386)	2,020	708	564	1,873	197
**West Virginia**												
No. of deaths (rate)	1,897 (98.0)	1,253 (134.8)	644 (62.6)	201 (33.9)	1,170 (125.0)	526 (166.2)	1,380 (71.1)	874 (95.2)	506 (47.9)	122 (20.6)	818 (88.5)	440 (140.5)
Costs, million USD (per capita, USD*)	2,149 (1,162)	1,599	530	369	1,618	94	1,507 (815)	1,099	393	225	1,133	77
**Wisconsin**												
No. of deaths (rate)	4,032 (64.2)	2,463 (85.0)	1,569 (43.7)	480 (24.1)	1,965 (64.8)	1,587 (174.2)	3,015 (46.7)	1,696 (58.4)	1,319 (35.1)	275 (13.8)	1,279 (41.6)	1,461 (159.6)
Costs, million USD (per capita, USD*)	3,934 (683)	2,895	967	906	2,617	229	2,499 (434)	1,765	700	508	1,665	203
**Wyoming**												
No. of deaths (rate)	514 (86.6)	355 (119.2)	159 (52.2)	81 (39.6)	322 (105.3)	111 (141.4)	361 (60.2)	234 (78.4)	127 (40.8)	46 (22.3)	225 (72.4)	90 (116.0)
Costs, million USD (per capita, USD*)	581 (995)	454	134	149^†^	415	21	384 (658)	286	103	83^†^	291	17

* Costs per capita calculated only for totals.

^†^ Average medical cost was statistically unstable; however, it accounted for less than 1% of combined average cost.

^§^ Rates based on ≤20 deaths might be unstable.

^¶^ Both average medical cost and average work loss cost were statistically unstable.

**FIGURE:**
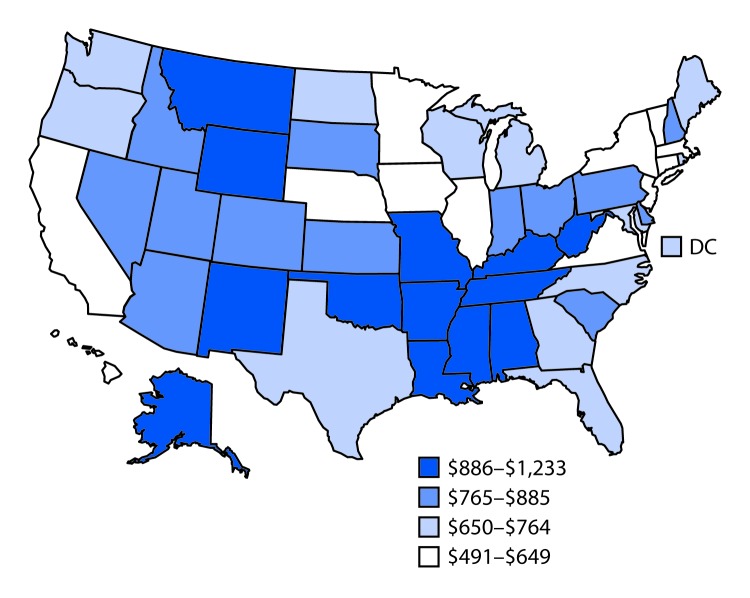
Costs per capita* of fatal injuries of all intents — United States, 2014 * In 2014 U.S. dollars.

## Unintentional Injuries

West Virginia had the highest lifetime costs per capita for fatal unintentional injuries ($815), the highest unintentional injury mortality rate among males (95.2), and the highest unintentional injury mortality rate among persons aged 25–64 years (88.5) (Table 1). Maryland had the lowest lifetime costs per capita for fatal unintentional injuries ($261), the lowest total unintentional injury mortality rate (26.4), the lowest male unintentional injury mortality rate (36.9), and the lowest unintentional injury mortality rate among persons aged 25–64 years (23.3). New Mexico had the highest total unintentional injury mortality rate (71.9) and the highest female unintentional injury mortality rate (49.9). California had the highest lifetime costs for fatal unintentional injuries ($12.2 billion) and the highest number of unintentional injury deaths (11,804).

## Suicides

Alaska and New Jersey had the highest and lowest lifetime suicide costs per capita ($338 and $107, respectively) (Table 2). Montana had the highest total suicide rate (23.8), the highest male suicide rate (36.8), and the highest female suicide rate (11.4). DC had the lowest number of suicides (52), total suicide rate (7.7), male suicide rate (12.3), and lifetime costs ($73 million). California had the highest lifetime costs ($4.9 billion) and the highest number of suicides (4,214).

**TABLE 2:** Suicide and homicide deaths, rates per 100,000 population, lifetime medical and work-loss costs, and lifetime medical and work-loss costs per capita, by state — United States, 2014

State	Suicides			Homicides		
	Total	Sex		Total	Sex	
		Male	Female		Male	Female
**Alabama**						
No. deaths (rate)	715 (14.5)	569 (24.3)	146 (5.6)	374 (8.0)	304 (13.4)	70 (2.8)
Costs, million USD (per capita, USD*)	897 (185)	755	143	606 (125)	532	76^†^
**Alaska**						
No. deaths (rate)	167 (22.0)	138 (34.8)	29 (7.9)	37 (4.7)	22 (5.3)	15 (4.1)
Costs, million USD (per capita, USD*)	249^†^ (338)	220^†^	32^†^	61^†^ (83)	38^†^	—**
**Arizona**						
No. deaths (rate)	1,244 (18.0)	945 (27.7)	299 (8.7)	322 (5.0)	249 (7.7)	73 (2.2)
Costs, million USD (per capita, USD*)	1,528 (227)	1,222	293	538 (80)	448	82^†^
**Arkansas**						
No. deaths (rate)	515 (17.2)	406 (27.9)	109 (7.2)	217 (7.6)	158 (11.3)	59 (4.1)
Costs, million USD (per capita, USD*)	671 (226)	550	119^†^	323 (109)	258	62^†^
**California**						
No. deaths (rate)	4,214 (10.5)	3,234 (16.7)	980 (4.7)	1,813 (4.6)	1,514 (7.6)	299 (1.5)
Costs, million USD (per capita, USD*)	4,927 (127)	3,986	933	3,103 (80)	2,794	337
**Colorado**						
No. deaths (rate)	1,083 (19.8)	843 (31.3)	240 (8.7)	177 (3.3)	124 (4.5)	53 (2.1)
Costs, million USD (per capita, USD*)	1,421 (265)	1,174	252	282 (53)	215	58^†^
**Connecticut**						
No. deaths (rate)	379 (9.7)	276 (14.8)	103 (5.1)	99 (2.8)	75 (4.3)	24 (1.3)
Costs, million USD (per capita, USD*)	475 (132)	368	98^†^	170 (47)	142	25^†^
**Delaware**						
No. deaths (rate)	126 (13.2)	100 (22.3)	26 (5.3)	57 (6.5)	47 (10.9)	10 (2.2)
Costs, million USD (per capita, USD*)	168^†^ (179)	140^†^	—**	98 (105)	87^†^	—**
**District of Columbia**						
No. deaths (rate)	52 (7.7)	39 (12.3)	13 (4.0)	97 (13.2)	79 (22.3)	18 (4.8)
Costs, million USD (per capita, USD*)	73^†^ (110)	59^†^	—**	180 (273)	152	—**
**Florida**						
No. deaths (rate)	3,035 (13.8)	2,328 (21.9)	707 (6.3)	1,158 (6.2)	915 (9.8)	243 (2.5)
Costs, million USD (per capita, USD*)	3,332 (167)	2,701	624	1,852 (93)	1,584	282
**Georgia**						
No. deaths (rate)	1,294 (12.6)	998 (20.6)	296 (5.6)	658 (6.5)	518 (10.2)	140 (2.8)
Costs, million USD (per capita, USD*)	1,622 (161)	1,323	292	1,087 (108)	933	155
**Hawaii**						
No. deaths (rate)	204 (13.6)	163 (21.5)	41 (5.4)	30 (2.3)	21 (3.0)	^††^
Costs, million USD (per capita, USD*)	283 (199)	243	43^†^	34^§^ (24)	—**	—**
**Idaho**						
No. deaths (rate)	320 (20.1)	240 (30.5)	80 (10.1)	36 (2.4)	22 (3.0)	14 (1.7)
Costs, million USD (per capita, USD*)	391^†^ (239)	299^†^	89^†^	49^§^ (30)	—**	—**
**Illinois**						
No. deaths (rate)	1,398 (10.4)	1,110 (17.1)	288 (4.2)	792 (6.2)	679 (10.6)	113 (1.8)
Costs, million USD (per capita, USD*)	1,780 (138)	1,474	304	1,409 (109)	1,307	123
**Indiana**						
No. deaths (rate)	948 (14.3)	756 (23.4)	192 (5.6)	364 (5.7)	290 (9.0)	74 (2.3)
Costs, million USD (per capita, USD*)	1,210 (183)	1,023	194	597 (90)	515	86^†^
**Iowa**						
No. deaths (rate)	407 (12.8)	327 (20.7)	80 (5.2)	78 (2.5)	50 (3.2)	28 (1.8)
Costs, million USD (per capita, USD*)	520 (167)	437	81^†^	114 (37)	87^†^	32^§^
**Kansas**						
No. deaths (rate)	455 (15.7)	356 (25.0)	99 (6.6)	104 (3.6)	75 (5.2)	29 (2.1)
Costs, million USD (per capita, USD*)	624 (215)	511	111^†^	168 (58)	132	34
**Kentucky**						
No. deaths (rate)	727 (15.9)	582 (26.2)	145 (6.2)	203 (4.7)	153 (7.1)	50 (2.3)
Costs, million USD (per capita, USD*)	927 (210)	771	151	303 (69)	253	55^†^
**Louisiana**						
No. deaths (rate)	679 (14.3)	506 (22.2)	173 (7.0)	538 (11.6)	428 (18.6)	110 (4.7)
Costs, million USD (per capita, USD*)	888 (191)	692	176	941 (202)	796	135
**Maine**						
No. deaths (rate)	220 (15.7)	174 (25.5)	46 (6.7)	23 (2.0)	15 (2.6)	—^††^
Costs, million USD (per capita, USD*)	269^†^ (202)	219^†^	49^†^	35^§^ (26)	—**	—**
**Maryland**						
No. deaths (rate)	606 (9.8)	470 (16.1)	136 (4.2)	387 (6.6)	312 (10.8)	75 (2.4)
Costs, million USD (per capita, USD*)	763 (128)	617	140^†^	692 (116)	593	91^†^
**Massachusetts**						
No. deaths (rate)	596 (8.3)	472 (13.6)	124 (3.4)	110 (1.6)	91 (2.7)	19 (0.5)
Costs, million USD (per capita, USD*)	782 (116)	657	126	197 (29)	176	24^†^
**Michigan**						
No. deaths (rate)	1,354 (13.2)	1,062 (21.3)	292 (5.6)	589 (6.2)	465 (9.8)	124 (2.6)
Costs, million USD (per capita, USD*)	1,735 (175)	1,461	276	990 (100)	831	149
**Minnesota**						
No. deaths (rate)	686 (12.2)	525 (18.8)	161 (5.9)	101 (1.9)	69 (2.6)	32 (1.2)
Costs, million USD (per capita, USD*)	914 (168)	741	172	170 (31)	125	40^†^
**Mississippi**						
No. deaths (rate)	380 (12.5)	299 (20.8)	81 (5.3)	332 (11.3)	277 (19.4)	55 (3.5)
Costs, million USD (per capita, USD*)	481 (161)	406	74^†^	530 (177)	484	62^†^
**Missouri**						
No. deaths (rate)	1,017 (16.3)	817 (27.2)	200 (6.3)	441 (7.5)	357 (12.3)	84 (2.8)
Costs, million USD (per capita, USD*)	1,302 (215)	1,091	205	745 (123)	650	94
**Montana**						
No. deaths (rate)	251 (23.8)	197 (36.8)	54 (11.4)	30 (2.9)	23 (4.4)	—^††^
Costs, million USD (per capita, USD*)	302^†^ (295)	250^†^	52^†^	40^†^ (39)	—**	—**
**Nebraska**						
No. deaths (rate)	251 (13.4)	202 (21.7)	49 (5.4)	63 (3.4)	47 (5.0)	16 (1.7)
Costs, million USD (per capita, USD*)	313 (166)	263	51^†^	108 (58)	91^†^	—**
**Nevada**						
No. deaths (rate)	573 (19.5)	449 (31.2)	124 (8.2)	176 (6.3)	138 (9.8)	38 (2.7)
Costs, million USD (per capita, USD*)	669 (236)	547	124^†^	266 (94)	235	41^§^
**New Hampshire**						
No. deaths (rate)	247 (17.6)	191 (27.5)	56 (8.1)	17 (1.3)^¶^	—^††^	—^††^
Costs, million USD (per capita, USD*)	302^†^ (228)	251^†^	49^†^	—**	—**	—**
**New Jersey**						
No. deaths (rate)	786 (8.3)	590 (12.9)	196 (4.1)	372 (4.4)	302 (7.2)	70 (1.6)
Costs, million USD (per capita, USD*)	958 (107)	748	203	654 (73)	568	80^†^
**New Mexico**						
No. deaths (rate)	449 (21.0)	350 (33.4)	99 (9.2)	135 (6.8)	106 (10.5)	29 (2.9)
Costs, million USD (per capita, USD*)	594 (285)	501	98	218 (105)	183	32^§^
**New York**						
No. deaths (rate)	1,700 (8.1)	1,262 (12.5)	438 (4.0)	662 (3.3)	536 (5.5)	126 (1.2)
Costs, million USD (per capita, USD*)	2,139 (108)	1,674	435	1,157 (59)	1,010	147
**North Carolina**						
No. deaths (rate)	1,351 (13.0)	984 (19.8)	367 (6.9)	551 (5.6)	435 (8.9)	116 (2.3)
Costs, million USD (per capita, USD*)	1,685 (169)	1,296	369	730 (73)	769	128
**North Dakota**						
No. deaths (rate)	137 (17.5)	113 (27.8)	24 (6.7)	15 (2.0)^¶^	13 (3.0)	—^††^
Costs, million USD (per capita, USD*)	195^†^ (264)	169^†^	—**	—**	—**	—**
**Ohio**						
No. deaths (rate)	1,491 (12.6)	1,163 (20.1)	328 (5.7)	578 (5.2)	472 (8.4)	106 (1.9)
Costs, million USD (per capita, USD*)	1,939 (167)	1,588	344	955 (82)	843	122
**Oklahoma**						
No. deaths (rate)	736 (19.1)	561 (29.5)	175 (9.2)	250 (6.5)	183 (9.5)	67 (3.5)
Costs, million USD (per capita, USD*)	999 (258)	801	186	409 (105)	316	83^†^
**Oregon**						
No. deaths (rate)	782 (18.7)	614 (30.1)	168 (7.9)	99 (2.4)	65 (3.1)	34 (1.7)
Costs, million USD (per capita, USD*)	911 (229)	755	157^†^	131 (33)	104^†^	33^†^
**Pennsylvania**						
No. deaths (rate)	1,817 (13.3)	1,440 (21.6)	377 (5.6)	620 (5.1)	492 (8.1)	128 (2.0)
Costs, million USD (per capita, USD*)	2,307 (180)	1,928	378	1,059 (83)	901	149
**Rhode Island**						
No. deaths (rate)	113 (10.0)	82 (14.9)	31 (5.4)	27 (2.5)	23 (4.2)	—^††^
Costs, million USD (per capita, USD*)	159^†^ (151)	120^†^	—**	45^†^ (43)	—**	—**
**South Carolina**						
No. deaths (rate)	753 (15.1)	579 (24.4)	174 (6.8)	363 (7.5)	286 (12.1)	77 (3.1)
Costs, million USD (per capita, USD*)	953 (197)	785	170	587 (121)	503	84^†^
**South Dakota**						
No. deaths (rate)	141 (17.0)	109 (25.9)	32 (7.9)	26 (3.2)	15 (3.6)	11 (2.7)
Costs, million USD (per capita, USD*)	197^†^ (231)	162^†^	37^†^	—**	—**	—**
**Tennessee**						
No. deaths (rate)	948 (14.1)	746 (23.3)	202 (5.8)	379 (5.9)	309 (9.6)	70 (2.2)
Costs, million USD (per capita, USD*)	1,241 (189)	1,032	214	595 (91)	523	82^†^
**Texas**						
No. deaths (rate)	3,254 (12.2)	2,528 (19.5)	726 (5.4)	1,389 (5.1)	1,059 (7.8)	330 (2.5)
Costs, million USD (per capita, USD*)	4,264 (158)	3,490	754	2,240 (83)	1,867	386
**Utah**						
No. deaths (rate)	559 (20.6)	418 (31.0)	141 (10.5)	61 (2.1)	39 (2.7)	22 (1.4)
Costs, million USD (per capita, USD*)	802 (273)	634	158^†^	89^†^ (30)	67^†^	25^†^
**Vermont**						
No. deaths (rate)	124 (18.6)	102 (30.7)	22 (7.2)	16 (2.9)^¶^	13 (4.8)	—^††^
Costs, million USD (per capita, USD*)	148^†^ (237)	131^†^	—**	—**	—**	—**
**Virginia**						
No. deaths (rate)	1,122 (12.9)	870 (20.7)	252 (5.7)	339 (4.1)	249 (5.9)	90 (2.2)
Costs, million USD (per capita, USD*)	1,412 (170)	1,150	252	555 (67)	449	105^†^
**Washington**						
No. deaths (rate)	1,119 (15.2)	854 (23.5)	265 (7.2)	211 (3.0)	157 (4.4)	54 (1.6)
Costs, million USD (per capita, USD*)	1,404 (199)	1,147	253	333 (47)	272	63^†^
**West Virginia**						
No. deaths (rate)	359 (18.1)	280 (28.6)	79 (8.1)	103 (5.9)	70 (7.9)	33 (3.9)
Costs, million USD (per capita, USD*)	426 (230)	346	71^†^	156 (85)	113^†^	41^†^
**Wisconsin**						
No. deaths (rate)	769 (13.1)	598 (20.6)	171 (5.9)	166 (3.0)	126 (4.5)	40 (1.4)
Costs, million USD (per capita, USD*)	981 (170)	806	170	274 (48)	227	45^†^
**Wyoming**						
No. deaths (rate)	120 (20.7)	96 (32.3)	24 (8.7)	24 (4.4)	16 (5.8)	—^††^
Costs, million USD (per capita, USD*)	153^†^ (262)	131^†^	21^†^	—**	—**	—**

* Costs per capita calculated only for totals.

^†^ Average medical cost was statistically unstable; however, it accounted for less than 1% of combined average cost.

^§^ Average medical cost was statistically unstable; however, it accounted for less than 5% of combined average cost.

^¶^ Rates based on ≤20 deaths might be unstable.

** Both average medical cost and average work loss cost were statistically unstable.

^††^ State-level counts and rates based on <10 deaths have been suppressed.

## Homicides

The highest and lowest lifetime homicide-related mortality costs per capita were in DC ($273) and Hawaii ($24), respectively (Table 2).^†^ DC had the highest total homicide rate (13.2), the highest male homicide rate (22.3), and the highest female homicide rate (4.8). New Hampshire, Maine, and Massachusetts had the lowest total homicide rate (1.3), the lowest male homicide rate (2.6), and the lowest female homicide rate (0.5), respectively. California had the highest lifetime homicide-related costs ($3.1 billion) and the highest number of homicides (1,813).

## Discussion

Economic burdens of fatal injuries varied widely in the 50 states and DC for each of the four categories of intent. Across all the four fatal injury intents, some states consistently had lower lifetime costs per capita than most other states. For example, New York, New Jersey, and California ranked among the five lowest states in terms of lifetime costs per capita for injuries of all intents, unintentional injuries, and suicides. In contrast, New Mexico ranked among the five highest states in terms of lifetime costs per capita for injuries of all intents, unintentional injuries, and suicides. Varying economic burdens of fatal injuries in the 50 states and DC might be attributed to the different injury mortality rates, the different medical costs resulting from different medical procedures, and the different demographic characteristics of injury decedents, such as sex and age.

Implementation of effective injury prevention strategies is needed to help reduce the substantial lifetime medical and work-loss costs associated with fatal injuries. The differing state-level lifetime costs per capita for fatal injuries suggests an urgent need in some states to prevent injuries. States that consistently have lower lifetime costs per capita across different intents of injuries might have successful injury prevention experiences that could be shared with states with higher per capita costs.

The findings in this report are subject to at least four limitations. First, the costs account for medical and work-loss costs associated with decedents. Other societal costs, such as criminal justice costs and the pain and suffering of family members, were not considered. Second, work-loss costs, based on the mean earnings of the general population by sex and specific age groups, might be over- or underestimated because the mean earnings of decedents might differ from those of the general population. Third, intent of fatal injury, as determined from the manner of death assigned on death certificates by coroners or medical examiners, might differ across jurisdictions (*5*). Finally, unintentional fatal injuries were not broken down into more specific categories such as motor vehicle crashes, drug overdoses, traumatic brain injuries, and older adult falls, so that this report cannot indicate the economic burdens of those specific categories of unintentional injuries.

During 2005–2014, the number of unintentional fatal injuries increased 15%, from 117,809 to 136,053, and unintentional injury moved from the fifth to the fourth leading cause of death; the number of suicides rose 31%, from 32,637 to 42,773, and suicide moved from the eleventh to the tenth leading cause of death (*2*,*6*). The increasing incidence and economic burden of injuries, particularly unintentional injuries and suicides, call for effective prevention programs and strategies. For example, the CDC Guideline for Prescribing Opioids for Chronic Pain provides prescribing recommendations for opioid pain medication to patients aged ≥18 years with chronic pain in primary care settings (*7*), which could be adopted by states and might reduce the number of persons who overdose prescribed opioid medications. To reduce motor vehicle crash fatalities, states could increase seatbelt use with primary enforcement seatbelt laws that cover everyone in the vehicle (*8*) or consider requiring car seats and booster seats for children through at least age 8 years or until seatbelts fit properly (*9*). The 2012 Surgeon General’s *National Strategy for Suicide Prevention* suggests that strategies enhancing social support, community connectedness, and access to mental health and preventive services and measures to reduce stigma and barriers associated with seeking help might alleviate suicide risk across the lifespan (*10*). The estimates of state-level economic burdens of fatal injuries will permit policy makers to compare the costs of implementing prevention programs and strategies with the cost savings garnered from the aversion of fatal injuries.

**Summary**

**What is already known about this topic?**

Injuries are a leading cause of death in the United States. Injury-associated deaths result in a substantial economic burden to the United States: the total estimated lifetime medical and work-loss costs were $214 billion in 2013. Injury and violence prevention strategies can save lives and reduce costs.

**What is added by this report?**

Lifetime costs and lifetime costs per capita were calculated for each of the 50 states and the District of Columbia (DC) and for each of four injury intent categories (all intents, unintentional, suicide, and homicide) for 2014. Economic burdens varied widely among the states and DC. Lifetime costs per capita ranged from $1,233 (New Mexico) to $491 (New York) among fatal injuries of all intents, from $815 (West Virginia) to $261 (Maryland) among unintentional injuries, from $338 (Alaska) to $107 (New Jersey) among suicides, and from $273 (DC) to $24 (Hawaii) for homicides.

**What are the implications for public health practice?**

States can engage more effectively and efficiently in injury prevention if they are aware of the economic burden of injuries, identify areas for immediate improvement, and devote necessary resources to those areas. States that consistently have lower lifetime costs per capita across different intents of injuries might have successful injury prevention experiences that could be shared with states with higher per capita costs.

## Footnotes

* All intents category includes legal intervention and undetermined intent of injury, in addition to unintentional, suicide, and homicide.

† Lifetime costs or lifetime costs per capita of homicides were not presented for New Hampshire, North Dakota, South Dakota, Vermont, and Wyoming because those states had unstable average medical and work loss costs or unstable homicide rates.
